# Cyclosporin A Disrupts Notch Signaling and Vascular Lumen Maintenance

**DOI:** 10.1371/journal.pone.0119279

**Published:** 2015-03-16

**Authors:** Raghav Pandey, Mark A. Botros, Benjamin A. Nacev, Allan R. Albig

**Affiliations:** 1 Department of Cancer and Cell Biology, University of Cincinnati, Cincinnati, Ohio, United States of America; 2 Department of Biology, Duke University, Durham, North Carolina, United States of America; 3 Department of Pharmacology and Molecular Sciences, and Medical Scientist Training Program, Johns Hopkins University School of Medicine, Baltimore, Maryland, United States of America; 4 Department of Biology, Boise State University, Boise, Idaho, United States of America; Emory University, UNITED STATES

## Abstract

Cyclosporin A (CSA) suppresses immune function by blocking the cyclophilin A and calcineurin/NFAT signaling pathways. In addition to immunosuppression, CSA has also been shown to have a wide range of effects in the cardiovascular system including disruption of heart valve development, smooth muscle cell proliferation, and angiogenesis inhibition. Circumstantial evidence has suggested that CSA might control Notch signaling which is also a potent regulator of cardiovascular function. Therefore, the goal of this project was to determine if CSA controls Notch and to dissect the molecular mechanism(s) by which CSA impacts cardiovascular homeostasis. We found that CSA blocked JAG1, but not Dll4 mediated Notch1 NICD cleavage in transfected 293T cells and decreased Notch signaling in zebrafish embryos. CSA suppression of Notch was linked to cyclophilin A but not calcineurin/NFAT inhibition since *N*-MeVal-4-CsA but not FK506 decreased Notch1 NICD cleavage. To examine the effect of CSA on vascular development and function, double transgenic Fli1-GFP/Gata1-RFP zebrafish embryos were treated with CSA and monitored for vasculogenesis, angiogenesis, and overall cardiovascular function. Vascular patterning was not obviously impacted by CSA treatment and contrary to the anti-angiogenic activity ascribed to CSA, angiogenic sprouting of ISV vessels was normal in CSA treated embryos. Most strikingly, CSA treated embryos exhibited a progressive decline in blood flow that was associated with eventual collapse of vascular luminal structures. Vascular collapse in zebrafish embryos was partially rescued by global Notch inhibition with DAPT suggesting that disruption of normal Notch signaling by CSA may be linked to vascular collapse. However, multiple signaling pathways likely cause the vascular collapse phenotype since both cyclophilin A and calcineurin/NFAT were required for normal vascular function. Collectively, these results show that CSA is a novel inhibitor of Notch signaling and vascular function in zebrafish embryos.

## Introduction

Cyclosporin A (CSA) is an immunosuppressant that binds to and suppresses cyclophilin A [1]. CSA binding to cyclophilin A not only inactivates cyclophilin A and other cyclophilin family members, but the cyclophilin/CSA complex also suppresses the calcineurin/NFAT signaling pathway [2]. Since calcineurin/NFAT signaling is important for the transcription of IL-2 and other pro-inflammatory proteins, it is via this mechanism that CSA gains its immunosuppressant activity [3, 4]. In addition to the cyclophilin A—NFAT/calcineurin signaling cascade, additional evidence also suggests that CSA may interact with the Notch signaling pathway. For example, in endothelial cells treated with CSA the Notch responsive HESR1 (Hey1) gene was increased more than any other analyzed gene [5]. Further evidence is provided by Mammucari et al who have discovered that integration of the Notch and NFAT/calcineurin signaling pathways seems to be important for keratinocyte differentiation [6] and Zanotti et al who have identified Notch and NFAT signaling as reciprocally inhibiting pathways that together regulate osteoblast function [7].

In addition to immunosuppression, CSA has also been shown to elicit a wide range of negative effects in the cardiovascular system including disruption of heart valve development, smooth muscle cell proliferation, and angiogenesis inhibition. In particular, CSA suppress angiogenesis in a variety of models including the chick CAM [8, 9], rat mesenteric-window [10], transplanted pancreatic islets [11], and finally in HUVEC endothelial cells cultured on Matrigel [12]. Despite these reports, the molecular mechanism by which CSA suppresses angiogenesis is poorly defined. Recently however, specific inactivation of calcineurin/NFAT with FK506 has been shown to suppress angiogenesis [13, 14] suggesting that CSA may block angiogenesis by indirectly blocking calcineurin activity only after first complexing with cyclophilin A. Contrary to this idea however, cyclophilin A has also been shown to regulate angiogenesis during inflammatory reactions [15] and a non-immunosuppressive analog of CSA (*N*-MeVal-4-CsA) that does not block calcineurin activity maintains anti-angiogenic activity suggesting that cyclophilin A rather than NFAT/calcineurin is linked to angiogenesis [16]. Based on these observations, significant controversy still exists about how CSA manipulates angiogenesis.

The original goal of this research was to investigate the ability of CSA to control Notch signaling, and to determine if CSA suppresses angiogenesis in zebrafish embryos. Our results show that CSA directly suppresses Notch signaling in response to Jagged1 but not Delta-like 4 and that CSA inhibition of cyclophilin A, but not calcineurin is linked to Notch inhibition. However, CSA did not appear to have a direct effect on angiogenesis in zebrafish embryos, but rather had widespread negative effects on cardiovascular function that were initiated by inhibition of both cyclophilin A and calcineurin.

## Results and Discussion

### CSA suppresses Notch signaling

**Fig 1:**
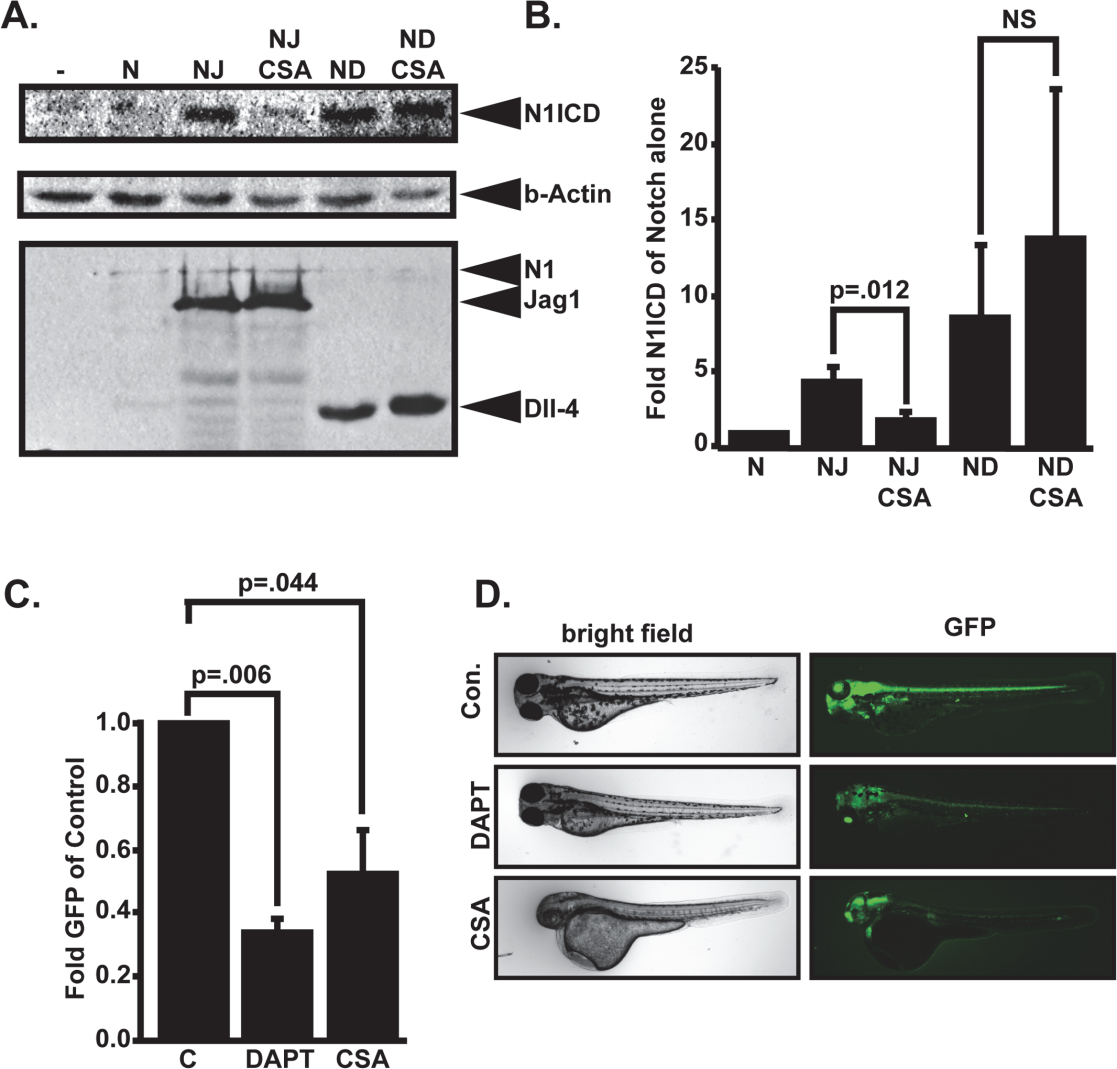
CSA blocks Notch signaling. (A) Effect of CSA on Notch signaling *in vitro*. 293T cells were transfected with various combinations of myc-tagged murine Notch1 (N), JAG1 (J), or Delta-like 4 (D) and treated with either 0.1% DMSO or 10μM CSA. Whole cell lysates were fractionated through SDS-PAGE gels and western blotted with anti-Val1744 antibody to detect cleaved Notch1 NICD fragments (N1ICD). Stripped blots were re-blotted with β-actin or 9E10 anti-myc antibodies to control for protein loading and expression of various transfected cDNAs. Shown are representative western blots from a single experiment that was performed five times in its entirety. (B) Western blot quantitation comparing N1ICD levels in cells transfected with Notch1 alone to cells transfected with combinations of Notch and JAG1 or Dll4 in the presence or absence of CSA. Displayed data represent the mean +/− SE of five individual experiments. P-values were calculated with the Student’s t-test. (C) Effects of CSA on Notch activity *in* vivo. Tp1bglob:eGFP embryos which express GFP from a tandem array of 12 Notch responsive RBP-Jk binding sites were incubated in either 0.1% DMSO, 10μM DAPT, or 10μM CSA. 48 hours later, GFP signal intensity was quantified in whole, live embryos. Data shown represents the mean +/− SE of 4 individual experiments. P-values were determined by student’s t-test. (D) Representative pictures of Tp1bglob:eGFP zebrafish embryos incubated with 10M DAPT or 10M CSA and imaged by fluorescent microscopy.

Previous results have suggested that CSA may interact with the Notch signaling pathway [5] although a molecular analysis of this interaction has not been performed. CSA is known to suppress signaling through the calcineurin/NFAT pathway [1] and interestingly, results from multiple labs suggests a functional association between calcineurin/NFAT and Notch [6, 7]. Based on these observations, we sought to determine if CSA controls Notch signaling. 293T cells were transfected with Myc-tagged versions of cDNAs encoding the murine Notch1 receptor either alone or in combination with the Notch ligands Jagged1 (JAG1) or Delta-like 4 (Dll4). Cyclosporine was applied 24 hours after transfection and Notch activation was monitored after overnight incubation by western blot analysis with antibodies that specifically detected the epitope generated by cleavage of Notch1 at Val1744 during the production of the active Notch1 N1ICD fragment. As shown in Fig. 1A, N1ICD levels were minimal in the absence of transfected Notch ligand and co-transfection with JAG1 or Dll4 successfully activated N1ICD cleavage above background levels. Interestingly, application of CSA blunted Notch N1ICD generation by JAG1 but not by Dll4. To confirm that CSA did not affect transfection efficiency or cDNA expression, we stripped the membranes and reblotted with anti-myc antibodies to detect myc-tags on the transfected Notch, JAG1, and Dll4 cDNAs. To control for differences in protein loading, equal volumes of cell lysates were blotted with anti β-actin antibodies. Fig. 1B summarizes this data by comparing N1ICD levels in cells transfected with Notch1 alone to cells transfected with combinations of Notch and JAG1 or Dll4 in the presence or absence of CSA treatment. Western blot data was quantified by densitometry, normalized to β-actin signal, and statistical analysis of the resulting data demonstrated that the CSA mediated decrease in JAG1—Notch signaling was significantly decreased while Dll4—Notch signaling was not significantly affected (Fig. 1B). These results indicated that CSA specifically blocks JAG1 but not Dll4 mediated N1ICD cleavage. The mechanistic basis for this observation is unknown, but these results argued against a mechanism involving differential expression of JAG1 or Dll4 protein. Instead, CSA could achieve this activity by modification of JAG1 or Dll4 ligand affinity for the Notch1 receptor, trafficking of JAG1 or Dll4 to the cell membrane, or one of several regulatory steps that control NICD accumulation in cells [17]. Finally, it will be interesting to determine if CSA also controls the activation of other Notch receptors such as Notch 3 that play important roles in vascular smooth muscle [18] and pericytes [19].

These results suggested that CSA decreases Notch signaling in transfected 293T cells, but it was important to determine if CSA also controls Notch signaling *in vivo*. To accomplish this, we monitored the affect of CSA on Notch activity in transgenic zebrafish expressing GFP from the Notch responsive TP1 element (i.e. Tp1bglob:eGFP) as previously described [20]. Freshly laid Zebrafish embryos were incubated in either 10μM CSA, an equivalent volume of DMSO, or 15μM gamma-secretase/Notch inhibitor DAPT as a positive control for reduced Notch signaling. After 24 hours, Notch activity in control and treated embryos was compared by measuring GFP fluorescence in a 96-well plate reader. Age matched non-fluorescent zebrafish embryos were also measured to establish a baseline of non-specific background fluorescence. As shown in Fig. 1C, both DAPT and CSA decreased GFP fluorescence indicating that both drugs suppressed Notch signaling. To visually confirm these results, fluorescence microscopy was used to qualitatively compare whole body GFP fluorescence in control, DAPT, or CSA treated embryos. As shown in Fig. 1D, compared to untreated embryos, there was a dramatic decrease in whole body GFP fluorescence in CSA and DAPT treated embryos. Collectively, these results demonstrated that CSA decreases Notch signaling both *in vitro* and *in vivo*.

### Cyclophilin A but not Calcineurin/NFAT controls Notch signaling

**Fig 2:**
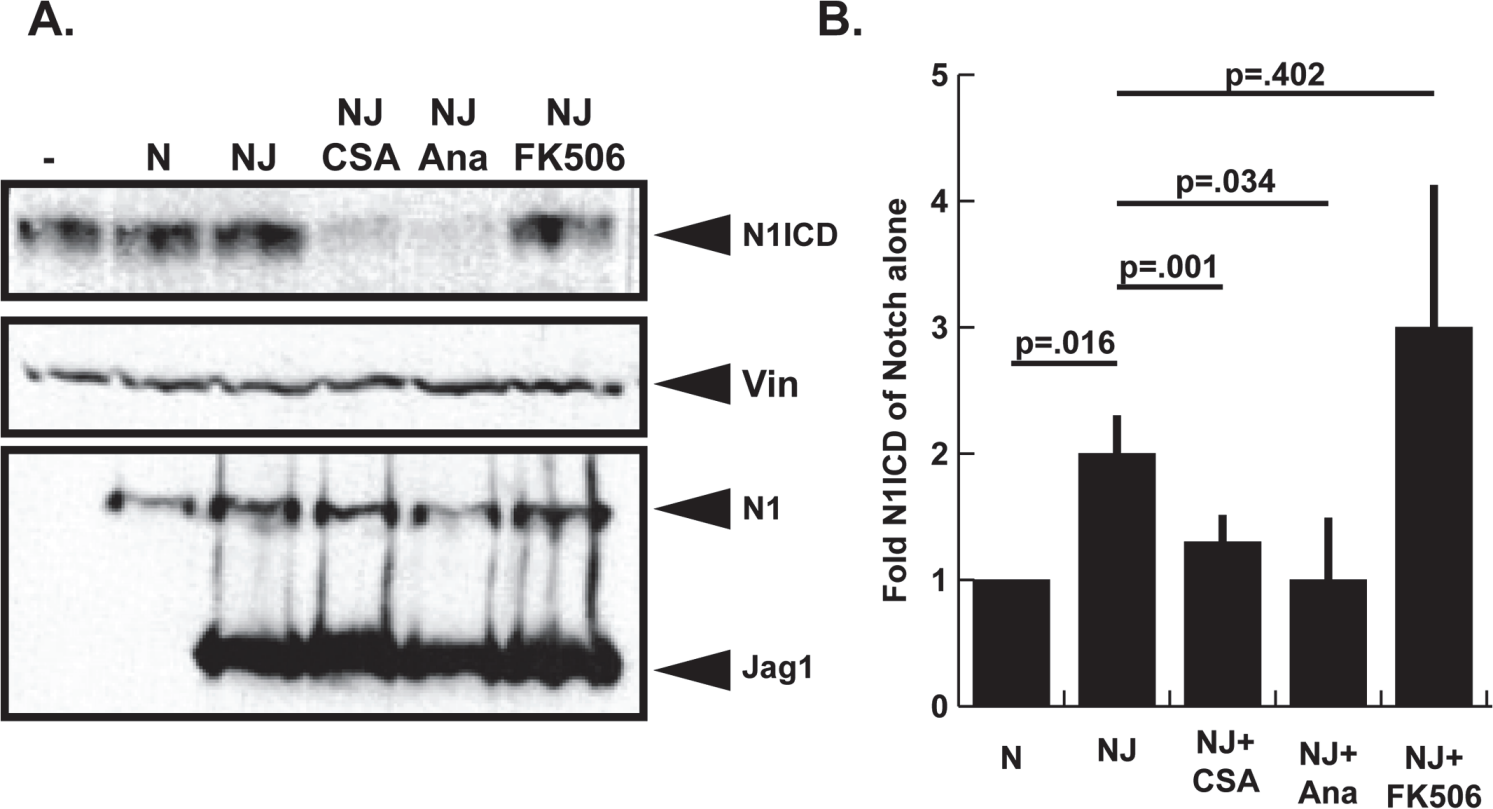
Inhibition of cyclophilin A but not calcineurin/NFAT reduces Notch signaling in 293T cells. (A) Effect of cyclophilin inhibition with N-MeVal-4-CsA analog, and calcineurin inhibition with FK506 on Notch signaling in 293T cells. 293T cells were transfected with either Notch1 (N) cDNA alone or Notch1 and JAG1 (NJ). The following day cells were treated with either 0.1% DMSO, 10μM CSA, 10μM *N*-MeVal-4-CsA (Ana), or 2μM FK506 for 24 hours. Whole cell lysates were fractionated through SDS-PAGE gels and blotted with anti-VAL1744 antibodies to detect cleaved Notch1 NICD fragment (N1ICD). Protein loading was monitored by stripping and reblotting membranes with anti-vinculin antibodies and equivalent cDNA expression was confirmed by reblotting with anti-Myc 9E10 antibodies. Shown is a representative result from experiments that were performed four times in their entirety. (B). Western blot quantitation comparing N1ICD levels in cells transfected with Notch1 alone to cells transfected with Notch1 and JAG1 in the presence or absence of CSA, *N*-MeVal-4-CsA, or FK506. Data shown represent the mean +/− SE of four experiments. P-values were determined using the Student’s t-test.

Binding of CSA to cyclophilin A not only inactivates cyclophilin A, but also forms a CSA/ cyclophilin A complex that subsequently deactivates calcineurin/NFAT function [2]. Since CSA suppresses activity of both cyclophilin A and calcineurin/NFAT, it was important to determine which pathway was functionally linked to CSA mediated Notch suppression. To accomplish this, we compared the Notch suppressing activity of the CSA analog *N*-MeVal-4-CsA which blocks cyclophilin A but not calcineurin/NFAT signaling [16], and tacrolimis (FK506) which inhibits calcineurin/NFAT but not cyclophilin A. 293T cells were again transfected with combinations of Notch1 and JAG1 then treated with solutions of 10μM CSA, 10μM CSA-analog, or 2μM FK506. As shown in Fig. 2A CSA-analog was able to suppress Notch-Jagged signaling in a similar manner to CSA, while FK506 was unable to block N1ICD accumulation. To control for differences in protein loading, the membrane was stripped and reblotted with anti-vinculin antibodies. To ensure equivalent expression of transfected Notch1 and JAG1 cDNA, membranes were stripped and reblotted with anti-Myc 9E10 antibodies to detect myc tags appended to the C-terminal of these proteins. Western blot data was quantified by densitometry, normalized to vinculin signal, and statistical analysis of the resulting data supported our conclusion that CSA and *N*-MeVal-4-CsA decreased JAG1—Notch signaling while FK506 did not significantly effect Notch signaling (Fig. 2B). The fact that CSA-analog, but not FK506 blocked JAG1—Notch1 signaling supported the idea that cyclophilin A, but not calcineurin/NFAT controls Notch signaling which is consistent with results from Shaw et al [5] showing that CSA but not FK506 controls HesR1 gene expression. This result however is inconsistent with other results [6, 7] that established connections between calcineurin/NFAT and Notch. Finally, although these experiments do not address the molecular mechanism whereby cyclophilin A controls Notch, it is interesting to note that prolyl isomerase activity helps fold the ankyrin domain of Notch NICD [21] and cyclophilin A (a prolyl isomerase) has been shown to accelerate folding of the ankyrin domain [22]. Moreover, another prolyl isomerase, PIN1 directly interacts with the NICD domain of Notch and regulates NICD cleavage and activation [23]. Therefore, it is tempting to speculate that inhibition of cyclophilin A (but not calcineurin/NFAT) may decrease NICD processing by interfering with NICD folding and processing.

### CSA causes vascular malfunction in zebrafish embryos

**Fig 3:**
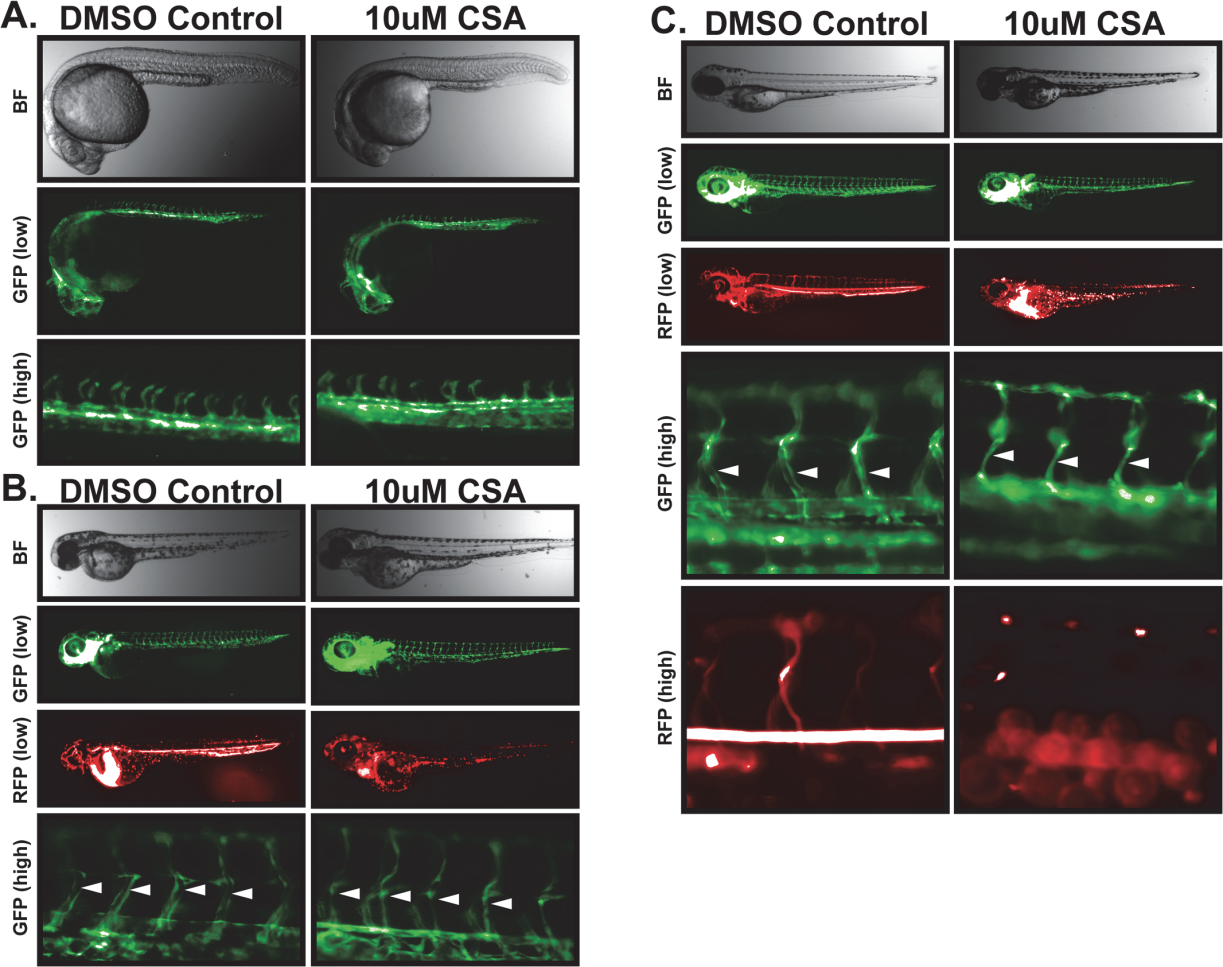
Cyclosporin-A destabilizes vascular lumen structures in zebrafish embryos. Freshly laid Fli1-GFP/GATA1-RFP zebrafish embryos were incubated in 10μM CSA or DMSO vehicle control for one, two, or four days. Whole embryo brightfield imaging was used to monitored gross morphology. Development of the vascular system was monitored by fluorescent microscopy of endothelial specific GFP expression. Circulatory flow was monitored by fluorescent microscopy of red-blood cell specific RFP expression. (A) Effects of CSA on 1dpf embryos. 1 day after CSA treatment, brightfield imaging of zebrafish embryos (top panel) was unable to distinguish any significant developmental impact of CSA on gross embryo morphology. Microangiogram analysis revealed similar development of the primitive vascular system including sprouting intersegmental vessels. (B) Effects of CSA on 2 dpf embryos. Zebrafish embryos treated with CSA for two days displayed no obvious signs of developmental abnormality in bright field images. Low power GFP imaging revealed an apparently normal vascular system, however RFP imaging revealed a distinct lack of blood flow throughout the embryo. High power GFP imaging revealed a lack of vascular lumen structures in ISV structures (arrows). (C) Effect of CSA on 4 dpf embryos. After four days of CSA treatment, no vascular luminal structures (arrows) or blood flow was evident in CSA treated embryos.

CSA treatment elicits a wide variety of effects on endothelial and smooth muscle cells in the vascular tree. Notch signaling has emerged as a major regulator in the vertebrate vascular system, serving roles in both endothelial (*i*.*e*. angiogenesis) and smooth muscle cells [24]. Given our results showing that CSA suppresses Notch signaling and the importance of Notch to vascular function, we set out to observe the effect of CSA treatment on angiogenesis and vascular function in zebrafish embryos. Freshly laid double transgenic Fli1-GFP / Gata1-RFP zebrafish embryos were incubated in solutions of 2–10 μM CSA, or DMSO vehicle for 1 to 4 dpf (days post fertilization). Vascular development was monitored by GFP imaging of endothelium while vascular function was monitored by RFP imaging of circulating blood cells in treated and control embryos. There were no obvious developmental defects in body morphology caused by 10μM CSA at any point from 1 to 4 dpf (Fig. 3A-C). Development of the aorta and cardinal vein also appeared normal after one day of CSA treatment (Fig. 3A). Contrary to the reported anti-angiogenic activity of CSA, initial sprouting of intersegmental vessels (ISV) from the aorta (Fig. 3A) was unaffected by 1 day of CSA treatment and the anastomosis of ISV vessels to form the dorsal lateral anastomotic vessel (DLAV) was also unaffected by CSA treatment after 2 days (Fig. 3B). Overall vascular patterning appeared normal in 2 dpf embryos (Fig. 3B GFP-low). However, while CSA treated embryos initially did have circulating blood cells and lumen structures, high power imaging of ISV vessels in embryos treated with CSA for 2 days revealed a progressive loss of luminal structure (Fig. 3B GFP-high) that was accompanied by a progressive loss of blood flow in ISV and aortic vessels and blood pooling near the heart (Fig. 3B RFP). Interestingly, CSA treatment of 2 dpf embryos with normal heart function and blood circulation also caused luminal collapse and loss of blood flow suggesting that the effect of CSA on vessel function may not be linked to initial heart development nor initial vascular development in the presence of CSA (data not shown). By 4 dpf, luminal structures in CSA treated zebrafish had collapsed entirely and blood flow was non-existent (Fig. 3C).

Collectively, these results showed that CSA does not appear to affect vasculogenesis or angiogenesis in zebrafish embryos since overall patterning and ISV sprouting was indistinguishable from control embryos. This is contradictory to several reports indicating that CSA is a negative regulator of angiogenesis [8, 10, 12, 16, 25]. However, these results do indicate that CSA has a major effect on subsequent maintenance of the vascular system. Unfortunately, these experiments did not have the power to dissect the ultimate cause of this dysfunction. Indeed, since Notch, cyclophilin A, and calcineurin/NFAT are involved in many aspects of the vascular system such as heart valve formation, lumen development/maintenance, and smooth muscle function, the observed vascular phenotype could be caused by disruption of any of these ubiquitous signaling mechanisms and lead to malfunction in a multitude of ways.

### Cyclophilin A and NFAT/calcineurin are both required for vascular function in zebrafish

**Fig 4:**
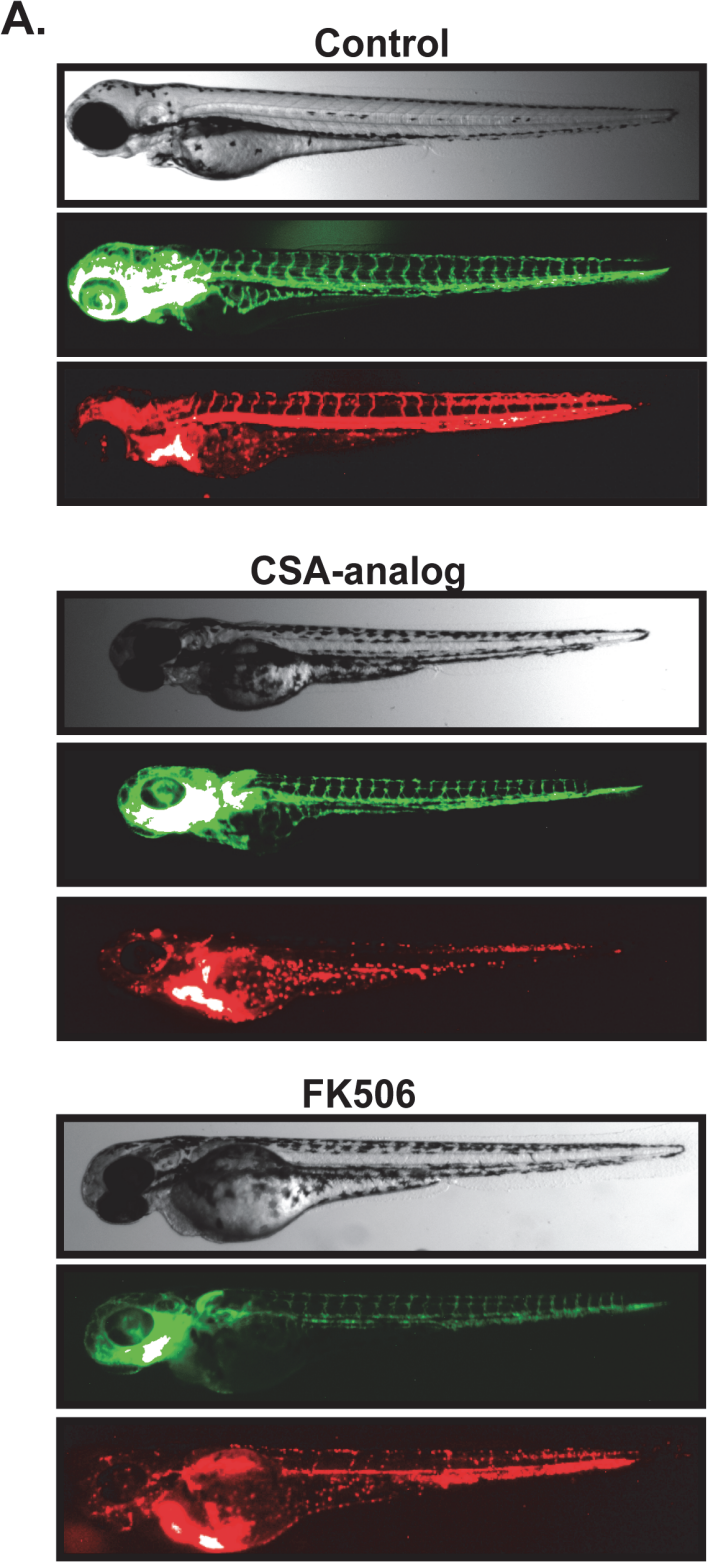
Inhibition of cyclophilin A or calcineurin/NFAT destabilizes lumen structure. (A) Freshly laid Fli1-GFP / GATA1-RFP embryos were incubated in 0.1% DMSO, 2μM FK506, or 40μM *N*-MeVal-4-CsA (CSA-Analog) for two days. Fluorescent imaging was used to monitor overall vascular development (GFP) and blood flow (RFP). Similar to CSA, neither FK506 nor *N*-MeVal-4-CSA had an impact on gross morphology or overall vascular development however both drugs blocked blood flow.

Previous results have shown that signaling through the calcineurin/NFAT pathway is required for endothelial response to VEGF [26], and for the normal development of smooth muscle cells and heart valves [27, 28]. Independently however, cyclophilin A has also been implicated in angiogenesis [16], regulation of VEGF signaling [29], and the development of smooth muscle cells and heart valves [15]. Since CSA suppresses both the calcineurin/NFAT and cyclophilin A signaling pathways, we attempted to discriminate which of these signaling pathways was linked to CSA induced vascular dysfunction. Freshly laid zebrafish embryos were treated with 1 to 10μM concentrations of FK506 to specifically inhibit calcineurin/NFAT and monitored for vascular collapse and loss of blood flow as before. Treatment of zebrafish embryos with 2μM or greater solutions of FK506 triggered a loss of blood flow through ISV and aortic vessels similar to 10 μM CSA treatment (Fig. 4). Specific inhibition of cyclophilin A with *N*-MeVal-4-CsA also caused a loss of vascular lumen structures and blood flow although 4-fold more (*i*.*e*. 40 μM) CSA analog was required for this effect.

These results suggested that both the calcineurin/NFAT and cyclophilin A pathways are required for proper maintenance of vascular lumen structures. Although both cyclophilin A and calcineurin/NFAT have been functionally linked to VEGF signaling in endothelial cells [26, 29], the observed vascular malfunction was not consistent with the effects of VEGF inhibition in zebrafish embryos. Indeed, treatment of zebrafish embryos with specific VEGF inhibitors including SU5416 [30] or PTK787 [31], or injection of anti-VEGF-A morpholinos [32] results in a dramatic loss of overall ISV development in zebrafish embryos. In contrast, we found that ISV vessels initially develop normally in CSA treated fish. Therefore, the phenotype caused by suppression of VEGF alone is not consistent with our results and suggests that CSA stimulated vascular dysfunction in zebrafish embryos does not involve inhibition of VEGF signaling.

### Global Notch suppression partially rescues vascular flow and lumen maintenance

**Fig 5:**
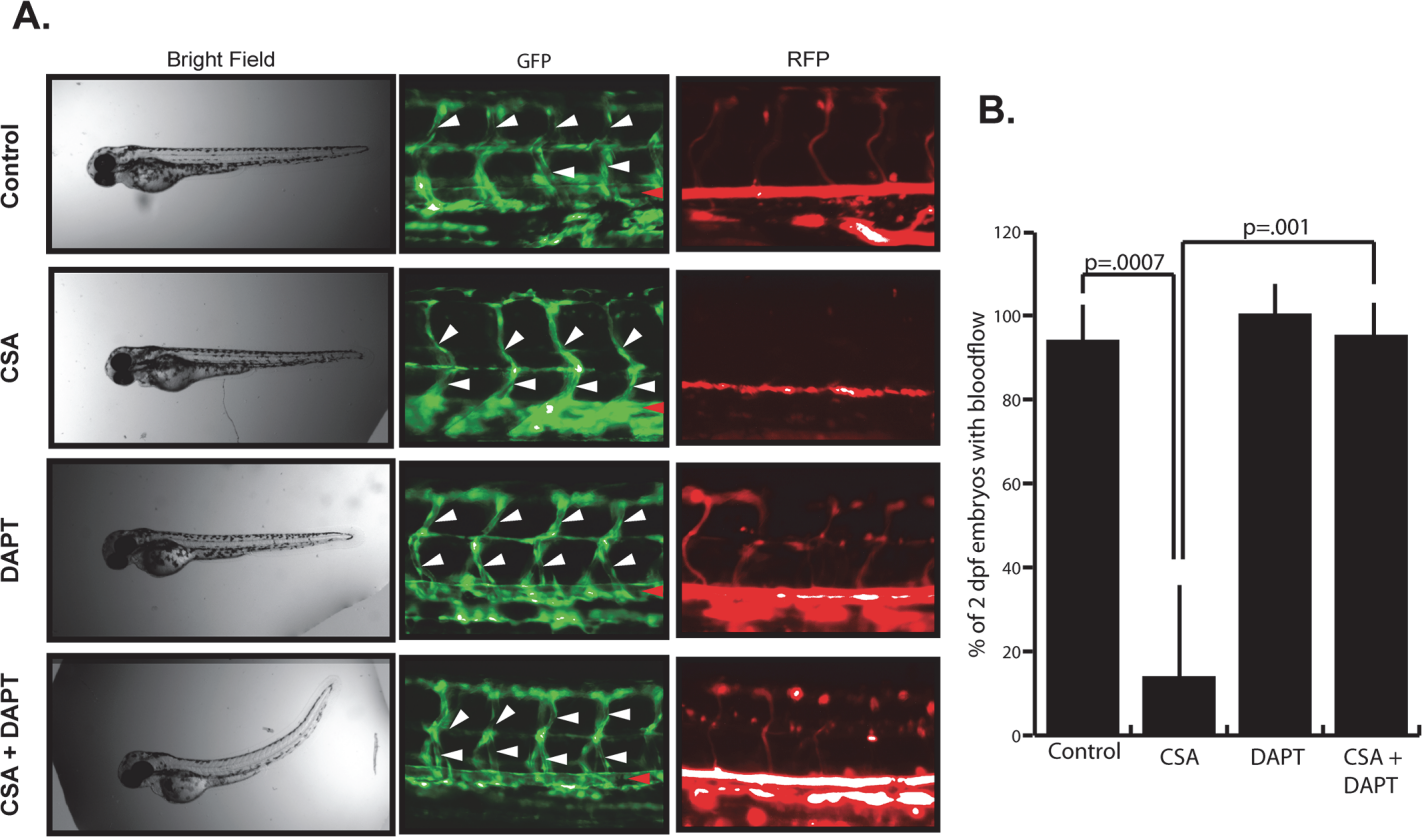
Notch inhibition partially rescues CSA induced vascular malfunction. (A) Effect of CSA and DAPT on vascular function in zebrafish embryos. Freshly laid Fli1-GFP / GATA1-RFP zebrafish embryos were incubated in 0.1% DMSO (Control), 10μM CSA, 15μM DAPT, or 10μM CSA + 15μM DAPT for 2 days. Bright field imaging revealed no gross morphological abnormalities in either CSA or DAPT treated fish, however CSA + DAPT treated fish experienced an acute curvature. GFP imaging revealed a lack of lumen structures in the ISV (white arrowheads) and aortic vessels (red arrowheads) of CSA treated fish. DAPT treated fish displayed normal luminal structure and blood flow. CSA + DAPT treated embryos had luminal structures (arrowheads) and blood flow similar to control or DAPT alone treated embryos. Shown are representative results from a single experiment that was performed five times in its entirety. (B) Quantitative analysis of blood flow in zebrafish treated with CSA, DAPT, or CSA + DAPT. Data shown represent the average +/− SE of five individual experiments. P-values were determined by student’s t-test.

Our results have shown that suppression of either cyclophilin A or calcineurin/NFAT leads to the vascular malfunction phenotype triggered by CSA treatment of zebrafish embryos. We also demonstrated that inhibition of cyclophilin A, but not calcineurin/NFAT decreased JAG1 (but not Dll4) mediated Notch activation in 293T cells. Based on these results, it was not clear if suppression of Notch was in any way functionally linked to the CSA vascular malfunction and we therefore attempted to determine if Notch suppression was implicated in this phenotype. We hypothesized that if the vascular defects were due solely to Notch inhibition, then suppression of Notch with the gamma secretase inhibitor drug DAPT should recapitulate the vascular defects induced by CSA treatment. As shown in Fig. 1C-D, 15μM DAPT reduced Notch-dependent GFP expression in zebrafish embryos to a similar level as 10μM CSA but this concentration was less than the 100μM solutions others have used to completely suppress Notch signaling in zebrafish embryos [20, 33, 34]. Therefore, treatment of zebrafish with this reduced DAPT concentration more accurately represented Notch suppression by CSA. As shown in Fig. 5, CSA again induced luminal collapse and a loss of blood flow in 2 dpf zebrafish embryos while 15μM DAPT alone did not appear to have any noticeable affect on vascular network development, lumen stability, or blood circulation. Thus, it did not appear that vascular dysfunction in CSA treated embryos was linked to simple Notch inhibition. However, bulk Notch activity alone is insufficient for proper vascular function. Instead, a balanced input from multiple Notch ligands such as JAG1 and Dll4 is critical for normal angiogenesis and vascular lumen formation [35]. Given that our earlier results suggested that CSA suppresses JAG1 but not Dll4, we hypothesized that CSA may disrupt vascular lumen maintenance by favoring Dll4 over JAG1 Notch signaling. Ideally, overexpression of JAG1 might have been used in an attempt to rescue the CSA induced vascular phenotype. However, given that our results in Fig. 2 suggested that CSA does not affect JAG1 expression, this approach did not seem appropriate. Instead, we rationalized that application of a low concentration of broad spectrum Notch inhibitor such as DAPT, might partially block both Dll4 and JAG1 to re-establish a rebalanced, albeit reduced activity of Notch signaling, and at least in part rescue luminal collapse and blood flow in CSA treated embryos. Freshly laid embryos were treated with 10μM CSA and 15μM DAPT then monitored for vascular collapse and blood flow after 2 days of treatment. As shown in Fig. 5A, DAPT partially prevented the collapse of vascular lumens and loss of blood flow in the ISV and aortic vessels of CSA treated fish. This rescue was not permanent however since fish treated with both CSA and DAPT eventually experienced vascular occlusion similar to that observed in CSA treated fish. Shown in Fig. 5B is a quantitative analysis of zebrafish embryos with blood flow through the aorta and at least one ISV vessel after 2 days of CSA, CSA+DAPT, or DAPT treatment. Interestingly, co-treatment with CSA and DAPT also elicited a striking curvature of the developing embryos suggesting an overlapping activity between CSA and Notch signaling. Collectively, these results demonstrated that the simple suppression of Notch by CSA was not likely to account for the vascular phenotype induced by CSA since DAPT alone was unable to recapitulate the effects of CSA on vascular function. Instead, these results suggest a more complex regulation of Notch by CSA that ultimately contributes to vascular malfunction. A more detailed analysis will need to be performed to fully understand the molecular mechanism by which CSA suppression of Notch contributes to vascular malfunction.

## Materials and Methods

### Ethics Statement

All work with zebrafish was approved by IACUC committee at Indiana State University and Boise State University and performed according to Indiana State University (Protocol #11–08–2007:AA) and Boise State University (Protocol # 006-AC13–004) Institutional IACUC guidelines.

### Transgenic Zebrafish

Transgenic (Tg(*fli-1*:eGFP)/Tg(*gata-1*:RFP) zebrafish were donated by Stephen C. Ekker (Mayo Clinic, Rochester MN). Freshly laid eggs were incubated in 10μM CSA, 15μM DAPT, or an equivalent concentration of DMSO and imaged 24–48 hpf under a fluorescent dissecting microscope (Nikon). Transgenic Notch reporter zebrafish (Tp1bglob:eGFP) were donated by Dr. Steven D. Leach (John Hopkins University School of Medicine, Baltimore, MD). Notch expression was monitored in 24 hpf embryos by measuring GFP fluorescence on a fluorescence multi-well plate reader.

### Cell culture and Transfection

293T Cells were grown in DMEM containing 10% FBS at 37°C with 5% CO_2_. 293T cells were transfected with 2μg of cDNAs encoding either myc-tagged Notch1, JAG1, of Dll4. 24 hours post transfection the cells were treated with either 10μM CSA, 2μM FK506, or 10μM DMSO and incubated for an additional 24 hours. The following day, cell lysates were prepared in boiling SDS-PAGE loading buffer and equal volumes were run on 10% SDS-PAGE gels.

### Western blotting

The VAL1744 polyclonal antibody (Cell Signaling Solutions) was used at a dilution of 1:250 to detect cleaved Notch1. The 9E10 monoclonal antibody (Santa Cruz) was used at a concentration of 1:1000 to detect myc-tagged Notch1, JAG1, and Dll4. The β-actin monoclonal antibody (Santa Cruz) was used at a dilution of 1:1000 to detect β-actin as a loading control. The anti-vinculin antibody (Santa Cruz) was used at a dilution of 1:1000

## References

[1] Clipstone NA, Crabtree GR. Calcineurin is a key signaling enzyme in T lymphocyte activation and the target of the immunosuppressive drugs cyclosporin A and FK506. Ann N Y Acad Sci. 1993;696:20–30. PubMed PMID: . 7509131 10.1111/j.1749-6632.1993.tb17138.x

[2] Liu J, Farmer JD Jr, Lane WS, Friedman J, Weissman I, Schreiber SL. Calcineurin is a common target of cyclophilin-cyclosporin A and FKBP-FK506 complexes. Cell. 1991;66(4):807–15. Epub 1991/09/02. PubMed PMID: . 1715244 10.1016/0092-8674(91)90124-h

[3] Welte K, Wang CY, Mertelsmann R, Venuta S, Feldman SP, Moore MA. Purification of human interleukin 2 to apparent homogeneity and its molecular heterogeneity. J Exp Med. 1982;156(2):454–64. Epub 1982/08/01. PubMed PMID: 6980256 10.1084/jem.156.2.454 PMC2186775

[4] Ruscetti FW, Gallo RC. Human T-lymphocyte growth factor: regulation of growth and function of T lymphocytes. Blood. 1981;57(3):379–94. Epub 1981/03/01. PubMed PMID: . 7006707

[5] Shah G, Middleton FA, Gentile KL, Tripathi S, Bruch D, Maier KG, et al Cyclosporine inhibition of angiogenesis involves the transcription factor HESR1. J Surg Res. 2008;149(2):171–6. PubMed PMID: 10.1016/j.jss.2008.03.016 18694572

[6] Mammucari C, Tommasi di Vignano A, Sharov AA, Neilson J, Havrda MC, Roop DR, et al Integration of Notch 1 and calcineurin/NFAT signaling pathways in keratinocyte growth and differentiation control. Dev Cell. 2005;8(5):665–76. PubMed PMID: . 15866158 10.1016/j.devcel.2005.02.016

[7] Zanotti S, Smerdel-Ramoya A, Canalis E. Reciprocal regulation of Notch and nuclear factor of activated T-cells (NFAT) c1 transactivation in osteoblasts. J Biol Chem. 2011;286(6):4576–88. PubMed PMID: 10.1074/jbc.M110.161893 21131365 PMC3039329

[8] Iurlaro M, Vacca A, Minischetti M, Ribatti D, Pellegrino A, Sardanelli A, et al Antiangiogenesis by cyclosporine. Exp Hematol. 1998;26(13):1215–22. PubMed PMID: . 9845377

[9] Ribatti D, Vacca A, Cantatore FP, Ria R, Benagiano V, Roncali L, et al An experimental study in the chick embryo chorioallantoic membrane of the anti-angiogenic activity of cyclosporine in rheumatoid arthritis versus osteoarthritis. Inflamm Res. 2000;49(8):418–23. PubMed PMID: . 11028759 10.1007/s000110050610

[10] Norrby K. Cyclosporine is angiostatic. Experientia. 1992;48(11–12):1135–8. PubMed PMID: . 1282107 10.1007/BF01948007

[11] Vajkoczy P, Vollmar B, Wolf B, Menger MD. Effects of cyclosporine A on the process of vascularization of freely transplanted islets of Langerhans. J Mol Med (Berl). 1999;77(1):111–4. PubMed PMID: . 9930941 10.1007/s001090050314

[12] Nacev BA, Liu JO. Synergistic inhibition of endothelial cell proliferation, tube formation, and sprouting by cyclosporin A and itraconazole. PLoS One. 2011;6(9):e24793 PubMed PMID: 10.1371/journal.pone.0024793 21969860 PMC3182171

[13] Courtwright A, Siamakpour-Reihani S, Arbiser JL, Banet N, Hilliard E, Fried L, et al Secreted frizzle-related protein 2 stimulates angiogenesis via a calcineurin/NFAT signaling pathway. Cancer Res. 2009;69(11):4621–8. PubMed PMID: 10.1158/0008-5472.CAN-08-3402 19458075 PMC2699405

[14] Siamakpour-Reihani S, Caster J, Bandhu Nepal D, Courtwright A, Hilliard E, Usary J, et al The role of calcineurin/NFAT in SFRP2 induced angiogenesis—a rationale for breast cancer treatment with the calcineurin inhibitor tacrolimus. PLoS One. 2011;6(6):e20412 PubMed PMID: 10.1371/journal.pone.0020412 21673995 PMC3108822

[15] Kim SH, Lessner SM, Sakurai Y, Galis ZS. Cyclophilin A as a novel biphasic mediator of endothelial activation and dysfunction. Am J Pathol. 2004;164(5):1567–74. PubMed PMID: . 15111303 10.1016/S0002-9440(10)63715-7 PMC1615642

[16] Nacev BA, Low WK, Huang Z, Su TT, Su Z, Alkuraya H, et al A calcineurin-independent mechanism of angiogenesis inhibition by a nonimmunosuppressive cyclosporin A analog. J Pharmacol Exp Ther. 2011;338(2):466–75. PubMed PMID: 10.1124/jpet.111.180851 21562139 PMC3141903

[17] Kadesch T. Notch signaling: the demise of elegant simplicity. Curr Opin Genet Dev. 2004;14(5):506–12. PubMed PMID: . 15380241 10.1016/j.gde.2004.07.007

[18] Ruchoux MM, Domenga V, Brulin P, Maciazek J, Limol S, Tournier-Lasserve E, et al Transgenic mice expressing mutant Notch3 develop vascular alterations characteristic of cerebral autosomal dominant arteriopathy with subcortical infarcts and leukoencephalopathy. Am J Pathol. 2003;162(1):329–42. Epub 2003/01/01. 10.1016/S0002-9440(10)63824-2 PubMed PMID: 12507916 PMC1851116

[19] Wang Y, Pan L, Moens CB, Appel B. Notch3 establishes brain vascular integrity by regulating pericyte number. Development. 2014;141(2):307–17. Epub 2013/12/07. 10.1242/dev.096107 PubMed PMID: 24306108 PMC3879812

[20] Parsons MJ, Pisharath H, Yusuff S, Moore JC, Siekmann AF, Lawson N, et al Notch-responsive cells initiate the secondary transition in larval zebrafish pancreas. Mech Dev. 2009;126(10):898–912. PubMed PMID: 10.1016/j.mod.2009.07.002 19595765 PMC3640481

[21] Bradley CM, Barrick D. Effect of multiple prolyl isomerization reactions on the stability and folding kinetics of the notch ankyrin domain: experiment and theory. Journal of molecular biology. 2005;352(2):253–65. Epub 2005/08/02. 10.1016/j.jmb.2005.06.041 PubMed PMID: . 16054647

[22] Bradley CM, Barrick D. The notch ankyrin domain folds via a discrete, centralized pathway. Structure. 2006;14(8):1303–12. Epub 2006/08/15. 10.1016/j.str.2006.06.013 PubMed PMID: . 16905104

[23] Rustighi A, Tiberi L, Soldano A, Napoli M, Nuciforo P, Rosato A, et al The prolyl-isomerase Pin1 is a Notch1 target that enhances Notch1 activation in cancer. Nat Cell Biol. 2009;11(2):133–42. Epub 2009/01/20. 10.1038/ncb1822 PubMed PMID: . 19151708

[24] Shawber CJ, Kitajewski J. Notch function in the vasculature: insights from zebrafish, mouse and man. Bioessays. 2004;26(3):225–34. PubMed PMID: . 14988924 10.1002/bies.20004

[25] Rafiee P, Heidemann J, Ogawa H, Johnson NA, Fisher PJ, Li MS, et al Cyclosporin A differentially inhibits multiple steps in VEGF induced angiogenesis in human microvascular endothelial cells through altered intracellular signaling. Cell Commun Signal. 2004;2(1):3 PubMed PMID: . 15175101 10.1186/1478-811X-2-3 PMC441414

[26] Zeini M, Hang CT, Lehrer-Graiwer J, Dao T, Zhou B, Chang CP. Spatial and temporal regulation of coronary vessel formation by calcineurin-NFAT signaling. Development. 2009;136(19):3335–45. PubMed PMID: 10.1242/dev.037903 19710169 PMC2739147

[27] Beis D, Bartman T, Jin SW, Scott IC, D'Amico LA, Ober EA, et al Genetic and cellular analyses of zebrafish atrioventricular cushion and valve development. Development. 2005;132(18):4193–204. Epub 2005/08/19. 10.1242/dev.01970 PubMed PMID: . 16107477

[28] Chang CP, Neilson JR, Bayle JH, Gestwicki JE, Kuo A, Stankunas K, et al A field of myocardial-endocardial NFAT signaling underlies heart valve morphogenesis. Cell. 2004;118(5):649–63. Epub 2004/09/02. 10.1016/j.cell.2004.08.010 PubMed PMID: . 15339668

[29] Alvarez-Arroyo MV, Yague S, Wenger RM, Pereira DS, Jimenez S, Gonzalez-Pacheco FR, et al Cyclophilin-mediated pathways in the effect of cyclosporin A on endothelial cells: role of vascular endothelial growth factor. Circ Res. 2002;91(3):202–9. Epub 2002/08/10. PubMed PMID: . 12169645 10.1161/01.res.0000027562.91075.56

[30] Cannon JE, Upton PD, Smith JC, Morrell NW. Intersegmental vessel formation in zebrafish: requirement for VEGF but not BMP signalling revealed by selective and non-selective BMP antagonists. Br J Pharmacol. 2010;161(1):140–9. PubMed PMID: 10.1111/j.1476-5381.2010.00871.x 20718746 PMC2962823

[31] Chan J, Bayliss PE, Wood JM, Roberts TM. Dissection of angiogenic signaling in zebrafish using a chemical genetic approach. Cancer Cell. 2002;1(3):257–67. PubMed PMID: . 12086862 10.1016/s1535-6108(02)00042-9

[32] Nasevicius A, Larson J, Ekker SC. Distinct requirements for zebrafish angiogenesis revealed by a VEGF-A morphant. Yeast. 2000;17(4):294–301. PubMed PMID: . 11119306 10.1002/1097-0061(200012)17:4<294::AID-YEA54>3.0.CO;2-5 PMC2448381

[33] Leslie JD, Ariza-McNaughton L, Bermange AL, McAdow R, Johnson SL, Lewis J. Endothelial signalling by the Notch ligand Delta-like 4 restricts angiogenesis. Development. 2007;134(5):839–44. PubMed PMID: . 17251261 10.1242/dev.003244

[34] Zecchin E, Filippi A, Biemar F, Tiso N, Pauls S, Ellertsdottir E, et al Distinct delta and jagged genes control sequential segregation of pancreatic cell types from precursor pools in zebrafish. Dev Biol. 2007;301(1):192–204. Epub 2006/10/25. 10.1016/j.ydbio.2006.09.041 PubMed PMID: . 17059815

[35] Alva JA, Iruela-Arispe ML. Notch signaling in vascular morphogenesis. Curr Opin Hematol. 2004;11(4):278–83. PubMed PMID: . 15314528 10.1097/01.moh.0000130309.44976.ad

